# Potential Application of *Saccharomyces cerevisiae* and *Rhizobium* Immobilized in Multi Walled Carbon Nanotubes to Adsorb Hexavalent Chromium

**DOI:** 10.1038/s41598-018-28067-9

**Published:** 2018-06-29

**Authors:** T. Sathvika, Amitesh Soni, Kriti Sharma, Malipeddi Praneeth, Manasi Mudaliyar, Vidya Rajesh, N. Rajesh

**Affiliations:** 1 Department of Chemistry, Birla Institute of Technology and Science, Pilani, Hyderabad campus, Jawahar Nagar, Hyderabad, 500 078 India; 2 Department of Biological Sciences, Birla Institute of Technology and Science, Pilani, Hyderabad campus, Jawahar Nagar, Hyderabad, 500 078 India

## Abstract

The presence of harmful contaminants in the waste stream is an important concern worldwide. The convergence of biotechnology and nanoscience offers a sustainable alternative in treating contaminated waters. Hexavalent chromium, being carcinogenic deserves effective and sustainable methods for sequestration. Here in, we report the immobilization of a prokaryote (*Rhizobium*) and eukaryote (*Saccharomyces cerevisiae*) in multiwalled carbon nanotubes (MWCNTs) for the effective adsorption of hexavalent chromium. The carboxylic groups were introduced into the MWCNTs during oxidation using potassium permanganate and were subjected to EDC-HOBT coupling to bind with microbial cell surface. FTIR, TGA, BET, FESEM-EDAX, HRTEM, XPS and confocal microscopy were the investigative techniques used to characterize the developed biosorbents. Experimental variables such as pH, adsorbent dosage, kinetics, isotherms and thermodynamics were investigated and it was observed that the system follows pseudo second order kinetics with a best fit for Langmuir isotherm. Electrostatic interactions between the functional groups in the microbial cell wall and hydrochromate anion at pH 2.0 propel the adsorption mechanism. The lab scale column studies were performed with higher volumes of the Cr(VI) contaminated water. Sodium hydroxide was used as the desorbing agent for reuse of the biosorbents. The sustainable biosorbents show prospects to treat chromium contaminated water.

## Introduction

Heavy metal contamination of water bodies leads to metal bioaccumulation in various forms, posing serious threat to the ecosystem. Heavy metals (Pb, Cr, As, Hg, Cd) are released into the water by various industrial activities thereby accumulating in food chain due to the binding ability with proteins causing deleterious effects in living beings^1^. Chromium is one of the widely used strategic metal in several industrial applications such as electroplating, leather tanning, and cement and catalyst production^2^. Chromium alloy coatings (CrAlSiN) are used in high speed machining applications since it enhances the metal resistant properties by encountering oxidation and corrosion^3^. Chromium exists in several oxidation states of which +3 and +6 are stable. Minimal amount of Cr(III) is required for glucose metabolism in the body whereas most of the Cr(VI) is a man-made carcinogen wherein its contamination is in limelight because of its cytotoxicity and genotoxicity^2,4^. Within the living cell, Cr(VI) undergoes reduction by producing various reaction intermediates such as Cr(V), Cr(IV), reactive oxygen species (ROS), peroxides, radicals and finally to Cr(III) which causes oxidative damage to DNA and affects DNA replication^5^. The USEPA^6^ has imposed stringent rules on the safe limit of total Cr in drinking water as 0.1 mg L^−1^. Several conventional methods such as precipitation, coagulation, ion exchange, adsorption, membrane filtration are used for treating metal contaminated water among which adsorption is the most effective and economical approach^7^. Therefore development of efficient adsorbents is necessary to remediate Cr(VI) being also important studying their reusability.

Chemical adsorbents developed to remediate Cr(VI) quite recently are xanthum gum grafted polyaniline – zinc oxide nanocomposite^8^, branched polyethylenimine grafted electrospunpolyacrylonitrile^9^, polyaniline coated titanate nanobelt^10^, MoS_2_/reduced graphene oxide^11^ and Fe_2_O_3_/graphitic C_3_N_4_ graphene nano composite^12^. Though the chemical based adsorbents show high adsorption capacity for removing heavy metals, their disposal is a major concern. Low cost and naturally available adsorbents such as biopolymers, agricultural wastes, microbes (bacteria/yeast/fungi) are used to remediate heavy metals as they are abundantly available^13^. The chitosan/graphene-oxide/montmorillonite composite for Cr(VI) adsorption developed by Yu *et al*.^14^ showed a very good adsorption capacity of 87.03 mg g^−1^. An agricultural corn bract waste was functionalised with polyethylenimine showed excellent performance for the removal and recovery of Cr(VI) from aqueous solutions^15^. *Pseudochrobactrum asaccharolyticum* isolated from Cr(VI) contaminated soils could completely remove 100 mg L^−1^ of hexavalent chromium in 144 hours^16^.

Microbes play a vital role in bioremediation of heavy metals by involving two mechanisms. A passive biosorption process takes place in living/non-living/dead microbes which is independent of microbe metabolism, and an active uptake which is a metabolism dependent process and occurs only in living microbes. Bioaccmulation is the process linking both active and passive modes of metal uptake^5,17^. The direct use of microbes in its native form for metal removal has several disadvantages such as poor mechanical strength, difficulty in microbe separation from the solution and there is always a possibility of loss of microbial mass after regeneration studies. To combat the disadvantages, microbes are immobilized in suitable matrices which help to improve the cell strength, rigidity, porosity and metal removal ability^18^. *Aspergillus BRVR* immobilized in montmorillonite and cellulose enhanced the Cr(VI) metal uptake capacity of microbes^19,20^, the exopolymeric substances (EPS) of *Pseudomonas* strains reduced majority of Cr(VI) to Cr(III) involving adsorption coupled reduction mechanism^21^, in an anaerobic sludge, Cr(VI) is reduced to Cr(III) by sulfidogenesis and also direct reduction of Cr(VI) was observed by chromate utilizing bacteria such as *Microbacterium*^22^. Therefore the possibility of utilizing microbes remain promising making the microbe immobilized matrices open for exploration as suitable biosorbents.

An eukaryotic unicellular microbe *Saccharomyces cerevisiae* (*S*.*cerevisiae*) commonly known as baker’s yeast is easily available and is known to have broad applications in food industry^23^ such as wine making, brewing, baking etc., As *S*. *cerevisiae* is classified under Generally Recognised as Safe (GRAS) product, it is widely used in industrial water treatment^24^. Sathvika *et al*.^25^ developed microbe based biosorbents with yeast immobilized in glutaraldehyde crosslinked cellulose matrix for the effective removal of Cr(VI) with a monolayer adsorption capacity of 23.61 mg g^−1^. Titania-yeast nanocomposite showed excellent potential of 99.2% removal for hexavalent chromium^23^.

A prokaryotic nitrogen fixing bacteria *Rhizobium* is a symbiotic, rod shaped, gram negative bacteria which is a potential biofertilizer for plants. It also helps in biological nitrogen fixation thereby influencing the agricultural productivity and is used in controlling root rot infections caused by fungi^26,27^. The legumes inoculated with *Rhizobium* inoculants is a common agriculture practice, which is a cost effective process to provide nutrients to the bacteria. Sludge generated from the agro based industries is rich in carbon and nitrogen sources which helps in the growth of *Rhizobium* thereby offering a green alternative to treat waste water as well as reducing the cost of inoculant preparation^28^. The activated biomass of *Rhizobium leguminosarum* could remove upto 77.3 ± 4.3% Cr(III) at 35 °C at pH 7.0^29^. An ND2 *Rhizobium* isolate from the root nodules of *Phaseolus vulgaris* turned out to be a potential biosorbent for Cr(VI) removal as well as promoting the growth properties of plant thereby increasing the agricultural productivity^30^. Understanding the potential of these two varied microbes is an interesting add-on for comparative analysis.

Nano- sized materials with their outstanding properties have attracted the attention of scientists and could efficiently be utilized for varied applications. The sp^2^ hybridized carbon nanotubes (CNT) is one of the interesting forms of elemental carbon categorized into two varieties. The first type is multiwalled carbon nanotubes (MWCNTs) which has concentric rings with a definite spacing between the layers and the second form is single walled carbon nanotubes (SWCNTs) which has a single layer cylinder. Significant progress has been made in the utilization of CNTs for the efficient removal of metal ions and pollutants as they possess large surface area, high mechanical strength, and excellent thermal, electrical properties and are small, hollow and layered structures^31^. Several MWCNTs adsorbents were developed for the treatment of aqueous solutions contaminated with Cr(VI). Lu *et al*.^32^ developed magnetic Fe_2_O_3_ nanoparticle-MWCNTs composite and was checked for the efficient removal of Cr(VI) at different temperatures with a q_max_ of 42.02 mg g^−1^ at 35 °C. Fe-Ag/f-MWCNT/PES Nanostructured-Hybrid Membranes developed by Masheane *et al*. could remove 94.8% of Cr(VI) from aqueous solution^33^. Chitosan was immobilized in nanoparticles and carbon nanotubes by forming a nanocomposite which could efficiently remove Cr(VI) up to 84% within a short contact time^34^. Reports involving microbe-MWCNTs combination for the sequestration of heavy metals are scarce. Yan *et al*.^35^ reported MWCNTs –calcium alginate complex immobilized in *Shewanella oneidensis* which showed higher reduction capacity of Cr(VI). *Pseudomonas aeruginosa* was immobilized in CNTs for effective adsorption of various heavy metals with q_max_ 6.60 mg g^−1^ for cobalt, 6.18 mg g^−1^ for cadmium, 6.07 mg g^−1^ for lead, 5.83 mg g^−1^ for manganese, 6.23 mg g^−1^ for chromium (III) and 5.25 mg g^−1^ for Ni^36^. Following a thorough literature review, we found there are no comparative studies on bacteria-MWCNTs and yeast-MWCNTs combination for sequestration of Cr(VI). In the current study, we demonstrate the differences in the efficiency of Cr(VI) uptake by an eukaryote (yeast) and a prokaryote (*Rhizobium*) immobilized in multiwalled carbon nanotubes. The pristine MWCNTs were oxidised and then were involved in coupling reaction with the amines present on the cell wall of microbes to form amide which react with hydrochromate ion and thus sequesters Cr(VI) from aqueous solutions. This would also pave way to explore the mechanistic and physiological variations leading to the differences in metal adsorption efficiencies.

## Results

### Molecular identification of the isolated microbial strains

**Figure 1:**
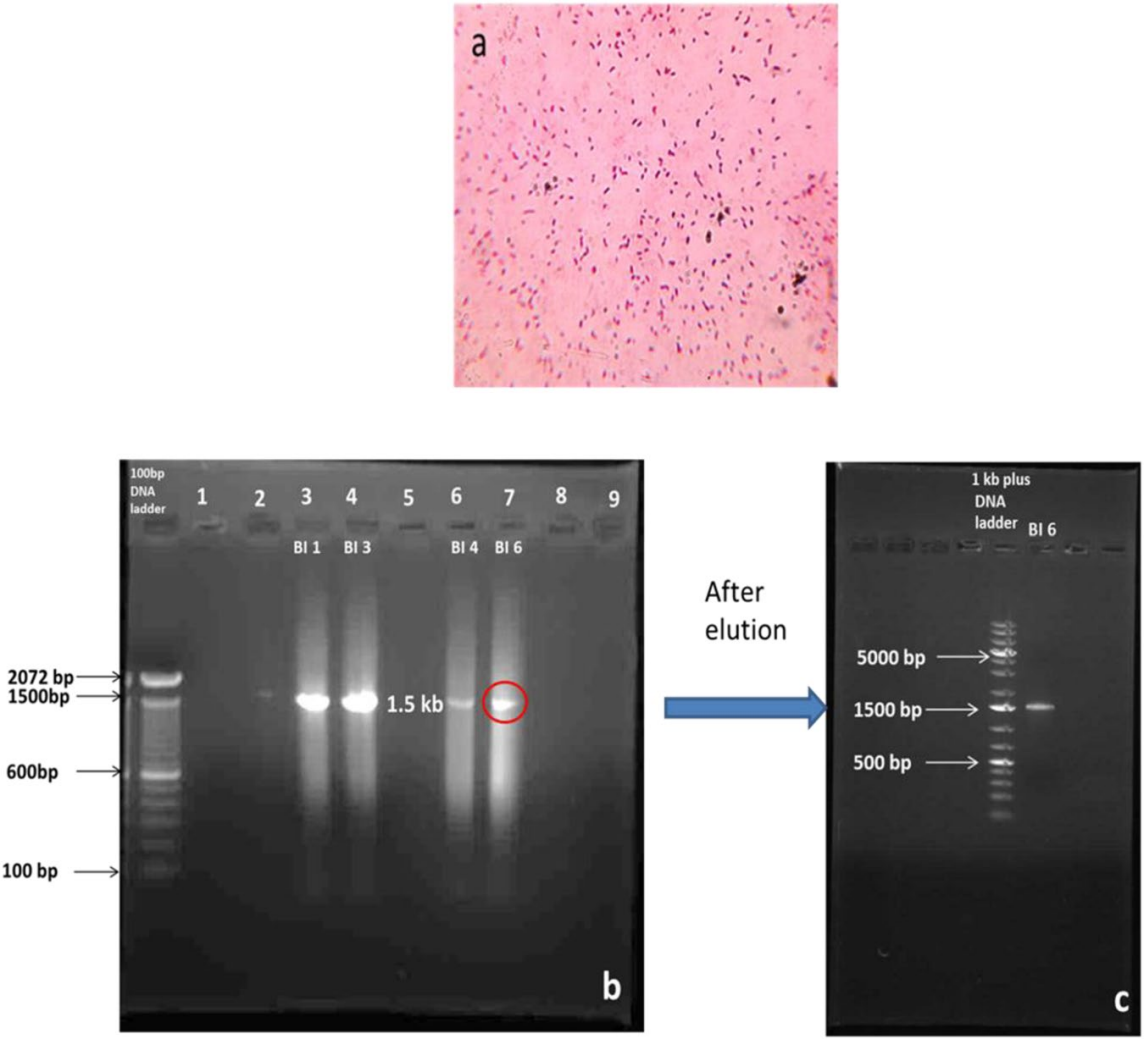
(**a**) Gram stain image of *Rhizobium BVR* (**b**) Gel picture of amplified products of 16S rDNA (1.5 kb) of the bacterial isolates; lane 1–100 bp DNA ladder Invitrogen (cat no: 15628050)]; 3 - bacterial isolate BI 1,; 4- bacterial isolate BI 3; 6 - bacterial isolate BI 4; 7- bacterial isolate BI 6. (**c**) Gel elution image of BI 6 [1 kb DNA ladder, Thermo scientific (cat no: SM1331)].

**Figure 2:**
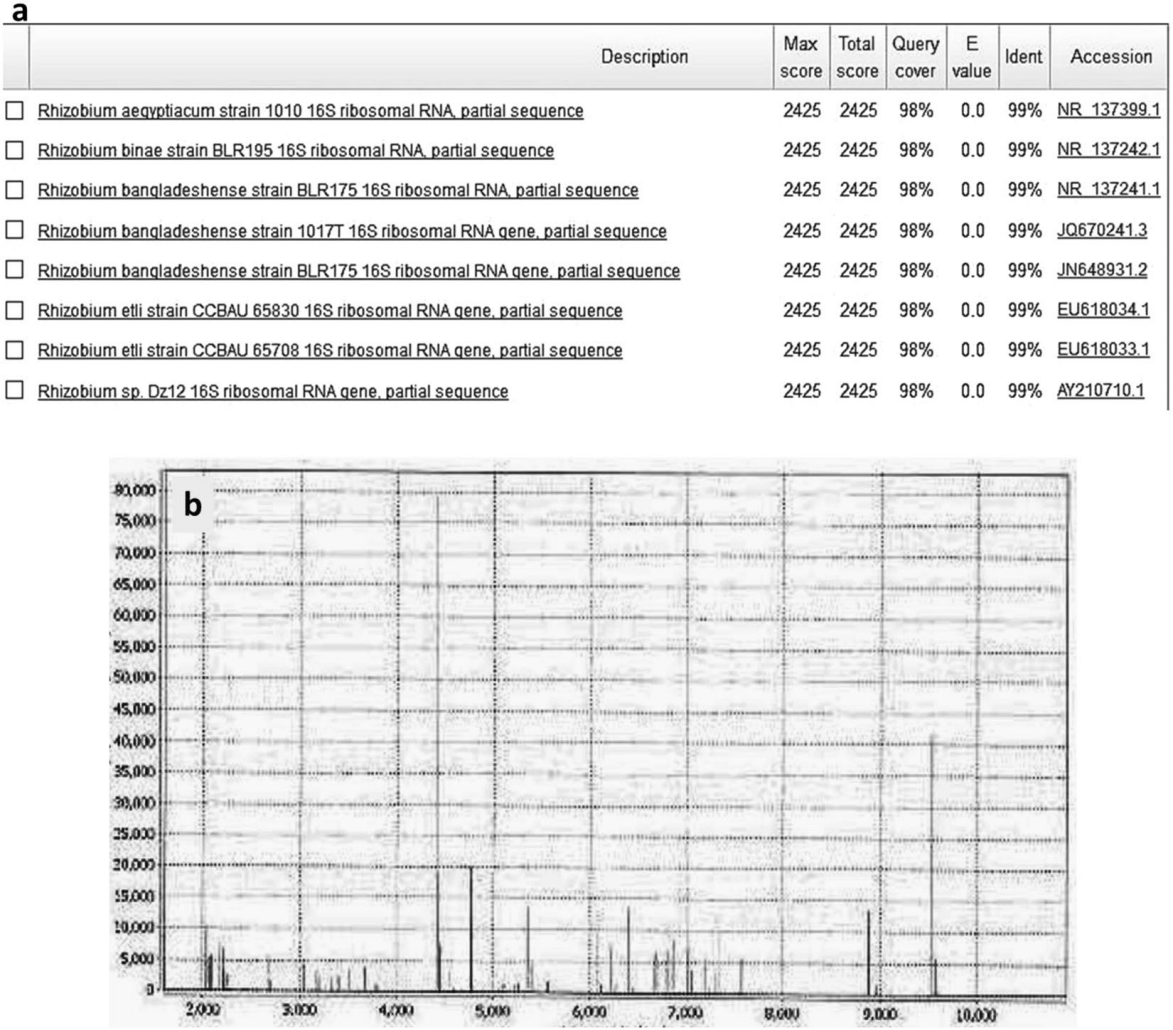
(**a**) Blast search for BI 6 sequence confirming *Rhizobium* species (**b**) MALDI TOF of *Rhizobium BVR*.

**Figure 3:**
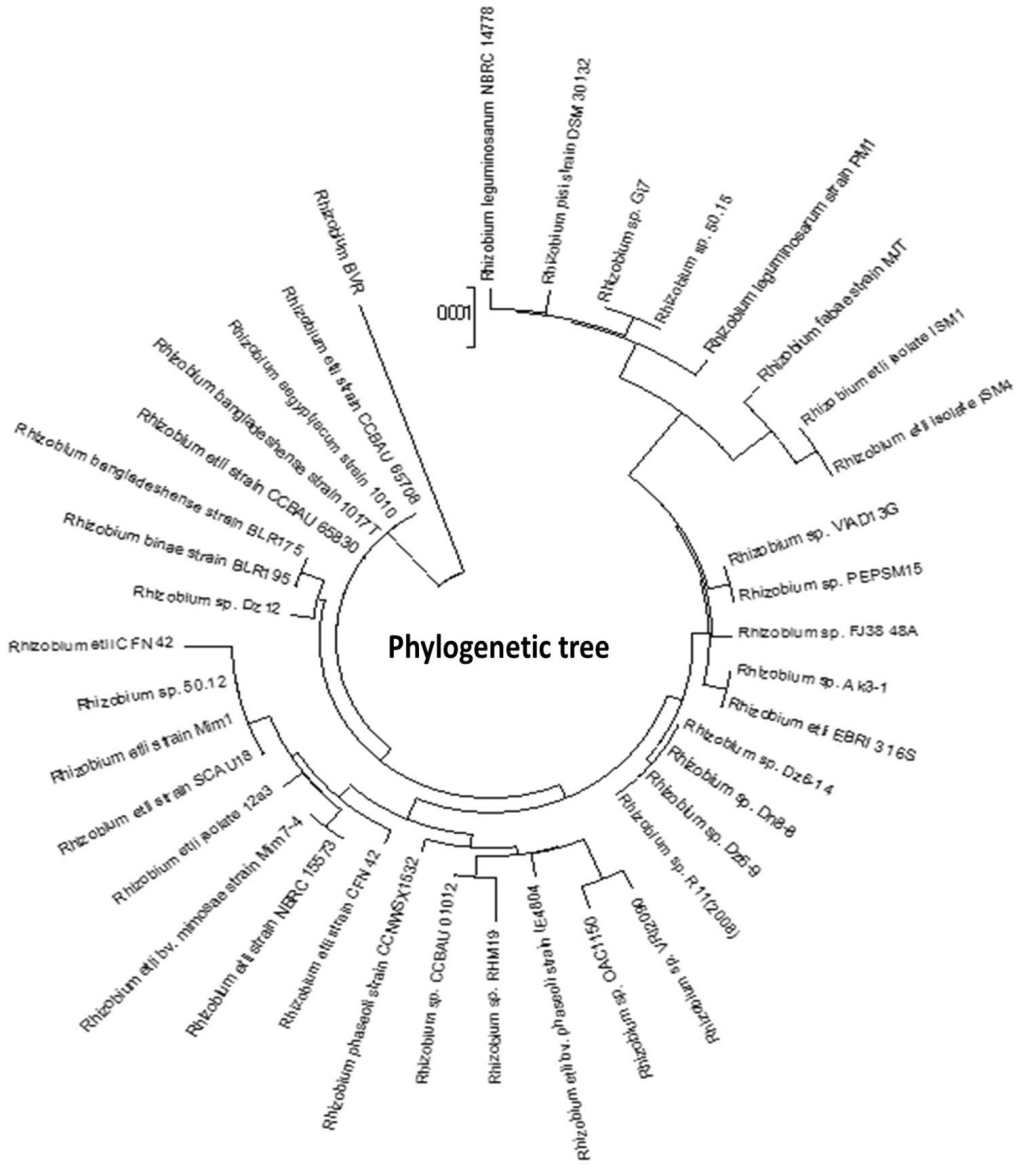
Phylogenetic tree of the *Rhizobium BVR* created using MEGA (version 6.0).

The morphological study of the gram stained bacteria (BI 1, 3, 4, 6) showed the presence of rod shaped (BI 4, 6) and cocco bacilli (BI 1, 3) gram negative bacteria (Fig. 1a). The isolated bacteria formed circular, translucent, white mucoid colonies on YMA Congo red medium which is a positive test for *Rhizobium*. Further biochemical tests were performed and the results of four isolates were tabulated in Table 1. 16S rDNA sequencing was performed to confirm the genus of the bacterial isolates with 1500 bp amplicon obtained after PCR (Fig. 1b,c). Among the sequences obtained, the database available in NCBI gene bank BI 6 strain showed 99% similarity to *Rhizobium* species (Fig. 2a) and thus bacterial isolate was named as *Rhizobium BVR* which was used for further study. The sequence was assigned an accession number MF136764 when submitted to NCBI Genbank. The evolutionary relationships as seen in the phylogenetic tree (Fig. 3) generated using MEGA 6.0 version showed the *Rhizobium BVR* is distantly related to other *Rhizobium* species. The MALDI TOF spectrum also confirmed the isolated strain to be *Rhizobium* (Fig. 2b). The confirmation of *S*.*cerevisiae* through the morphological and biochemical tests was reported in our previous studies^25^.

**Table 1:** Morphological and biochemical characteristics of the bacterial isolates.

Strain	Shape	Motility	Gram test	Indole	Methyl red	Voges-Prokasver	Citrate	Starch
BI1	Cocco bacillus	Motile	Gram negative	**+**	−	−	−	**+**
BI3	Cocco bacillus	Motile	Gram negative	**+**	−	−	−	**+**
BI4	Rods	Motile	Gram negative	−	−	−	**+**	−
BI6	Rods	Motile	Gram negative	−	−	−	**+**	−

### Characterisation of the biosorbent

**Figure 4:**
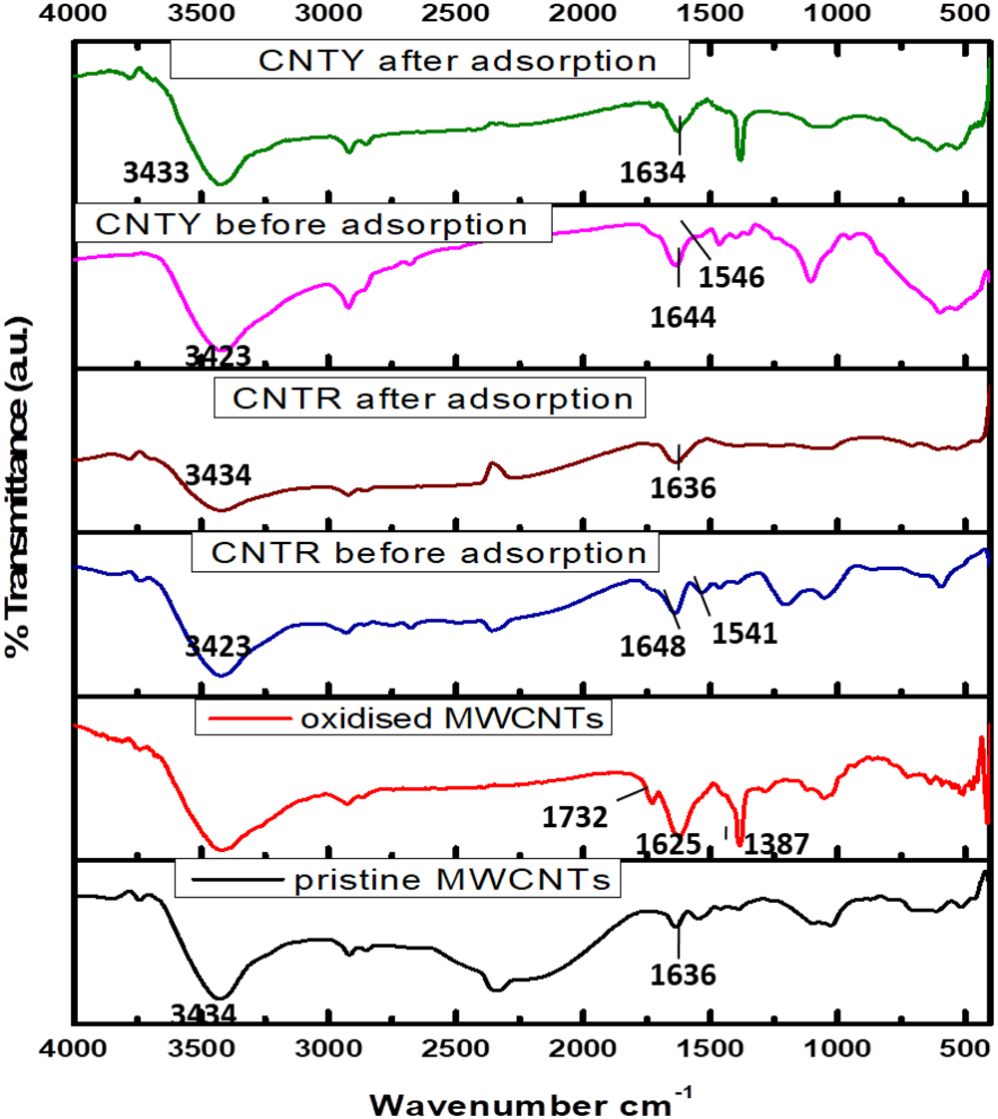
FTIR spectra of pristine, oxidised MWCNTs, before and after Cr(VI) adsorption on CNTR, CNTY.

The FTIR spectra of pristine and oxidised CNTs, biosorbents CNTR (multiwalled carbon nanotubes-*Rhizobium*) and CNTY (multiwalled carbon nanotubes-yeast) before and after Cr(VI) adsorption were recorded (Fig. 4). The pristine MWCNTs was oxidised using KMnO_4_ and H_2_SO_4_. The peak at 1732 cm^−1^corresponds to C=O of carboxylic acid confirming the introduction of COOH groups on the surface of MWCNTs. The carboxyl groups fluctuations leads to comparatively broader O-H stretch than in pristine CNTs. Symmetric and asymmetric COO^−^ stretchings^37^ gives rise to two peaks at 1387 cm^−1^ and 1625 cm^−1^. The successful immobilization of microbe in MWCNTs is assisted by EDC-HOBT coupling by forming amide bond around 1644–1648 cm^−1^ and also this peak is due to amide-1 of protein-peptide bond from the microbes^38^. The disappearance of carboxyl C=O peak after the amide formation indicates the involvement of carboxyl groups present on the surface of MWCNTs in amide formation. The band in the range 1541–1546 cm^−1^ corresponds to amide-II of in plane N-H bending^39^. After Cr(VI) adsorption the changes in the amide bond, O-H and C=O wavenumbers indicate they were involved in Cr(VI) uptake by protonating in acidic medium thereby forming electrostatic interactions with Cr(VI). The field emission scanning electron microscope (FESEM) and high resolution scanning electron microscopy (HRTEM) images of pristine, oxidised MWCNT and the biosorbent before and after Cr(VI) adsorption were recorded. The tiny lumps in FESEM images (Fig. S1c,d) and the particulates on MWCNTs in HRTEM images (Fig. S2c) indicate microbial immobilizations. The oxidation process caused minimal irregularities in the wall surfaces of MWCNTs^37^ as indicated in Fig. S2. The elemental analysis of the biosorbent before and after adsorption of Cr(VI) was recorded using Energy Dispersive X-ray Spectroscopy indicating Cr(VI) adsorption onto the biosorbent with characteristic peak between 5–6 keV (Fig. S1i–iv).

**Figure 5:**
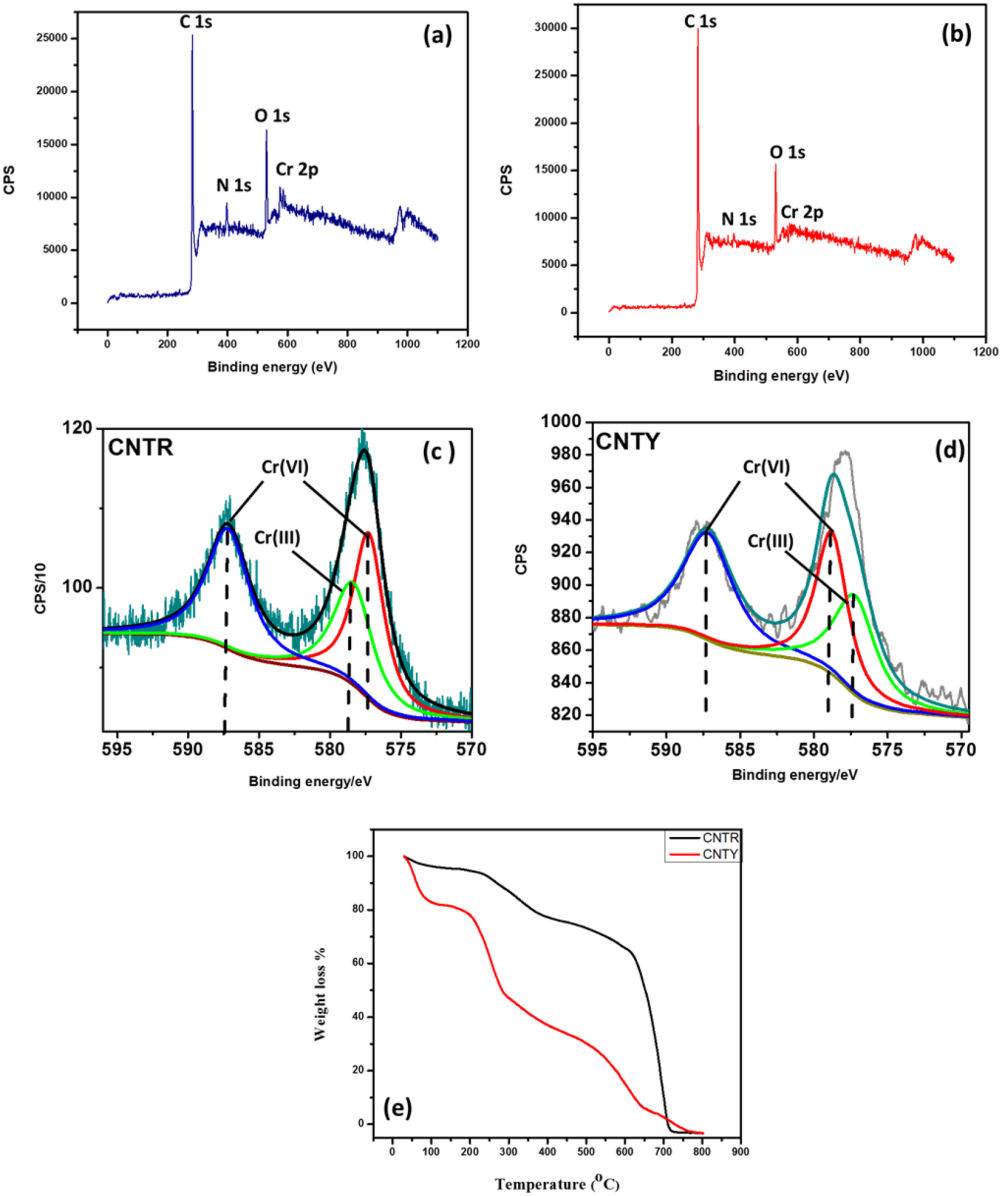
XPS spectra of (**a**,**b**) survey scan of CNTR and CNTY (**c**,**d**) high resolution chromium scan spectra (**e**) TGA of the biosorbents.

The presence of Cr(VI) on the biosorbent surface was also strongly supported by the X-ray photo electron spectroscopy (XPS). The base peak was corrected to 284.8 eV in the high resolution carbon scan. The survey scan of the biosorbents confirmed the presence of C, N, O and Cr respectively. The high resolution spectra of Cr 2p gave two peaks Cr2p_3/2_ and Cr2p_1/2_. Cr2p_3/2_ is deconvoluted into two peaks at 577 eV, 578 eV which represent Cr(III) and Cr(VI) respectively^40^ and Cr2p_1/2_ at 587 eV corresponds to Cr(VI) in both CNTR and CNTY shown in Fig. 5. Instant reduction of Cr(VI) was not observed after treatment with the biosorbents due to short agitation period and this was confirmed through chromium speciation by ion chromatography. The XPS analysis showed the presence of Cr(III) and as reported earlier^25^ Cr(III) formation on the biosorbent surface was evident only after 4–5 days. This could be due to extended interactions of Cr(VI) with the carbon and specific iron regulated surface proteins in the microbes^41,42^. The Brunauer–Emmett–Teller (BET) adsorption isotherm was used to measure the specific surface area of the biosorbent^43^. The nitrogen adsorption/desorption curves provided by the BET isotherm gave the surface areas for oxidised MWCNTs as 115.72 m^2^ g^−1^, 69.81 m^2^ g^−1^ for CNTR and 37.029 m^2^ g^−1^ for CNTY. The average pore volume and pore diameter was found to be as follows: Oxidised CNTs (1.3857 cm^3^ g^−1^, 47.897 nm), CNTR (0.9183 cm^3^ g^−1^, 52.613 nm) and CNTY (0.6573 cm^3^ g^−1^, 71.0 nm) respectively. These features could be attributed to the difference in the morphological features of the respective microorganisms. The thermal stability of the biosorbents were studied using thermogravimetric analysis (TGA). A sample mass of 2.43 mg of CNTR and 5.569 mg of CNTY were analyzed in air atmosphere at a flow rate of 50 mL min^−1^ in the temperature range 35–800 °C ramped at 10 °C per minute. The TGA curves (Fig. 5e) signify that biosorbents were stable at higher temperatures i.e., till 600 °C (CNTY) and 677 °C (CNTR) and the initial loss of mass is due to the moisture present in biosorbents^44^.

The presence of chromium in its +3 and +6 form were also differentiated using laser confocal microscopy using specific rhodamine based chemosensors. The physical properties of RBH such as colorless, non-fluorescent nature owes to its spirolactam structure which is highly stable and detects Cr(VI) whereas RF is a pale pink solid which exhibits fluorescence and detects Cr(III). RBH was dissolved in 10 mmol L^−1^ H_2_SO_4_ and added to the sample for further detection of Cr(VI). It was observed that after addition of RBH to the sample containing Cr(VI) it turned pink due to the conversion of RBH to RB in view of RBH oxidation caused by Cr(VI) in acid medium^45^. The excitation and emission was recorded at 560 nm and 585 nm respectively. RF when dissolved in 10 mmol L^−1^ Tris-HCl is a colorless solution. The RF was added to the biosorbent with Cr(VI) which did not show any fluorescence indicating no immediate reduction of Cr(VI). After 4 days, the addition of biosorbent to RF turned pink due to chelation of RF with Cr(III) present on the surface of the biosorbent, generating a rhodamine type product in spirolactam by ring opening at C-N bond^46^. The excitation and emission for Cr(III) in RF was recorded at 525 nm and 590 nm respectively. The bright field and fluorescent images of CNTR and CNTY are shown in Figs 3a–h and S3 i–viii. The images captured before and after addition of RBH confirmed the presence of Cr(VI) in sample and also Cr(III) presence was confirmed by the addition of RF which exhibited fluorescence.

### Effect of pH, adsorbent dosage and interaction mechanisms

**Figure 6:**
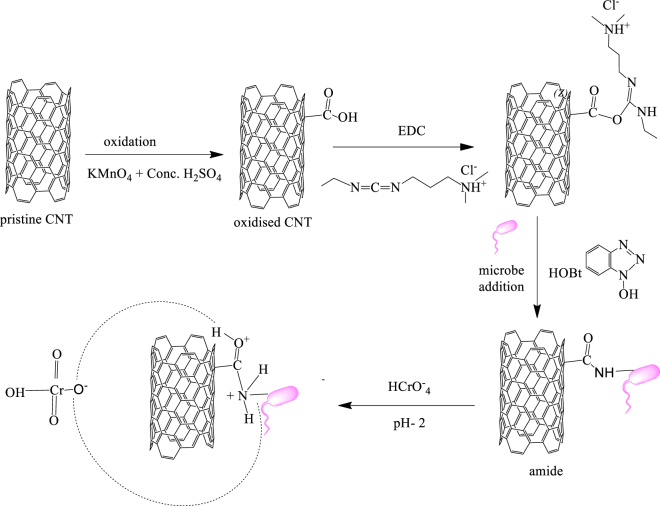
Interaction mechanism of the biosorbents with Cr(VI).

Influence of pH plays a vital role in the uptake of Cr(VI) onto the biosorbent. A 0.1 g weight of each of the biosorbent was weighed in a series of Erlenmeyer flasks and to that 20 mL of 5 mg L^−1^ Cr(VI) solution was added and adjusted to pH 2.0–7.0 and agitated for 3 hours to attain equilibrium. After analysis, it was found that the biosorbents could equally adsorb hexavalent chromium completely at pH 2.0. At pH > 2 it was observed that there was a decrease in the metal uptake (Fig. S4a). This is due to the existence of Cr(VI) ions in various forms such as hydro chromate (HCrO_4_^−^) at pH 2–4, in strongly acidic medium (pH < 2) it exists as dichromate (Cr_2_O_7_^2−^) and at higher pH as chromate (CrO_4_^2−^). In acidic medium, the biosorbent surface which has functional groups such as hydroxyl, carboxylic and amide gets protonated and is involved in electrostatic interactions with HCrO_4_^−^ thus aiding the metal to participate in biosorption (Fig. 6). Amides are usually poor leaving groups hence under highly acidic conditions (pH 2.0) the carbonyl oxygen of amide is protonated and further the protonation of amide nitrogen is also probable^47^ in influencing the metal uptake. According to HSAB (hard-soft acid base) principle CrO_4_^2−^ < HCrO_4_^−^ < OH^−^ with regard to the hardness of the ions^25^. At higher pH, the hydroxide and chromate ions compete each other resulting in the electrostatic repulsion thereby decreasing the Cr(VI) uptake. The adsorbent dosages were varied from 0.01 g to 0.5 g to observe the minimal dosage of the biosorbent which can remediate maximum amount of Cr(VI) at pH 2.0. A 20 mL volume of 5 mg L^−1^ Cr(VI) was taken with varying biosorbent dosage and agitated for 180 min and observed that with 0.1 g, CNTR as well as CNTY could adsorb Cr(VI) quantitatively indicating the saturation of adsorbent sites (Fig. S4b).

### Biosorption kinetics, isotherms and temperature effect studies

The kinetic experiments were performed using 0.1 g of the biosorbent mixed with 20 mL of 5 mg L^−1^ Cr(VI) at time intervals ranging from 5–180 min. The maximum uptake of the metal ion was observed at 180 min. The data obtained from the plots (Fig. S5a–c) were fitted into the pseudo first order^48^, second order^49^ and intra particle diffusion to evaluate the adsorption kinetics. The kinetic parameters are presented in Table S1. The equations representing pseudo first order and pseudo second order kinetics are given as

1 $$\mathrm{log}({{\rm{q}}}_{{\rm{e}}}-{{\rm{q}}}_{{\rm{t}}})=\,\mathrm{log}\,{{\rm{q}}}_{{\rm{e}}}-\frac{{{\rm{k}}}_{1}{\rm{t}}}{2.303}$$

2 $$\frac{{\rm{t}}}{{{\rm{q}}}_{{\rm{t}}}}=\frac{1}{{{\rm{k}}}_{2}{{\rm{q}}}_{{\rm{e}}}^{2}}+\frac{{\rm{t}}}{{{\rm{q}}}_{{\rm{t}}}}$$

The best suited kinetics depends on the experimental data which has the highest regression coefficient and the system follows pseudo second order kinetics with 1.844 mg g^−1^ (q_eexp_), 1.8848 mg g^−1^ (q_ecal_) for CNTY and 1.8026 mg g^−1^ (q_eexp_), 1.826 mg g^−1^ (q_ecal_) for CNTR. The ability of biosorbents to eliminate a unit mass of contaminants under similar conditions is explained by the isotherms. The plot q_t_ vs t^1/2^ relates to the intraparticle diffusion which is evaluated using q_t_ = k_i_ t^1/2^ + C where c corresponds to intercept and k_i_ is the intra particle diffusion constant which is obtained from the slope of the plot. The rate at which Cr(VI) gets adsorbed is mostly influenced by the diffusion mechanisms such as (i) the Cr(VI) from the bulk of the solution gets transferred onto the CNTR-CNTY biosorbent surface via external mass transfer (ii) intraparticle diffusion^50^ wherein Cr(VI) diffuses through the pores of the CNTR-CNTY biosorbent. From the plot it was observed that the straight line deviates from the origin having a significant intercept indicating the boundary layer phenomenon also plays role in the adsorption kinetics of hexavalent chromium.

Among several isotherms, commonly studied theoretical and empirical isotherms are Langmuir and Freundlich^51^. The data obtained from the plots (Fig. S5d,e) and the equations are presented in Table 2. The system assumed a monolayer Langmuir adsorption model as it has the low chi square value and high R^2^ value. The CNTY biosorbent has an adsorption capacity of 31.605 mg g^−1^ with an R^2^ value as 0.96 whereas CNTR has an adsorption capacity of 24.8 mg g^−1^ with an R^2^ value of 0.96. The dimensionless constant R_L_ value lies below unity indicating the reversibility of the isotherm which is represented as

3 $${{\rm{R}}}_{{\rm{L}}}=1/1+{{\rm{bC}}}_{{\rm{o}}}$$

where b is the Langmuir constant associated to adsorption energy, C_o_ represents the equilibrium concentration of the heavy metal ion. The Freundlich constants n and K_F_ are related to adsorption intensity and its capacity respectively. The values of n of both the biosorbents are greater than 1 (2.17, 2.29) indicating the favorability of metal adsorption onto the biosorbent. The pristine MWCNTs have an adsorption capacity of 11.93 mg g^−1^, when oxidised the uptake capacity enhanced to 16.22 mg g^−1^ and after the addition of *Rhizobium* and yeast to the MWCNTs adsorption capacity increased to 24.82 mg g^−1^ and 31.6 mg g^−1^ respectively.

**Table 2:** Biosorption isotherm parameters for Cr(VI) adsorption.

Langmuir$\frac{{{\boldsymbol{C}}}_{{\boldsymbol{e}}}}{{{\boldsymbol{q}}}_{{\boldsymbol{e}}}}{\boldsymbol{=}}\,\frac{{\bf{1}}}{{{\boldsymbol{q}}}_{{\boldsymbol{o}}}{\boldsymbol{b}}}{\boldsymbol{+}}\frac{{{\boldsymbol{C}}}_{{\boldsymbol{e}}}}{{{\boldsymbol{q}}}_{{\boldsymbol{o}}}}$	q_o_(mg g^−1^)	b (mg^−1^ L)	R_L_	R^2^	χ^2^
CNTY	31.605	0.072	0.578	0.967	1.021
CNTR	24.82	0.043	0.695	0.962	0.46
**Freundlich** ${\bf{log}}\,{{\boldsymbol{q}}}_{{\boldsymbol{e}}}=\,{\bf{log}}\,{{\boldsymbol{K}}}_{{\boldsymbol{F}}}-\frac{{\bf{1}}}{{\boldsymbol{n}}}\,{\bf{log}}\,{{\boldsymbol{C}}}_{{\boldsymbol{e}}}$	**K**_**F**_ **(mg** ^**1-1/n**^ **g** ^**−1**^ **L** ^**1/n**^ **)**	**n**	**R** ^**2**^	**χ** ^**2**^	
CNTY	3.298	2.2925	0.854	1.667	
CNTR	2.172	2.178	0.895	0.61	

The thermodynamic parameters such as Gibbs free energy (ΔG°), enthalpy (ΔH°) and entropy (ΔS°) explain the spontaneity of adsorption process. The equilibrium constant K at different temperatures was derived from the ratio of Cr(VI) present on the surface of CNTR, CNTY to that in the liquid phase and fitted into Gibbs free energy equation (ΔG = −RTlnK). The changes in enthalpy and entropy for the respective biosorbents were obtained from the Van’t Hoff plot of ln K against 1/T (Fig. S5f). The negative free energy values indicate the spontaneity in the biosorbent- sorbate interactions and the negative values of enthalpy and activation energy (E_a_ = ΔH°_ads_ + RT) indicates the system involves exothermic adsorption. The negative ΔS° values indicate the decrease in the disorderliness of the system with increase in temperature^25^. The ΔH values obtained for CNTR (−31.6 kJ mol^−1^) and CNTY (−72.2 kJ mol^−1^) indicate both the systems involve exothermic physicochemical adsorption^19^. The enthalpy-entropy compensation is well illustrated through the corresponding values obtained for the biosorbents. The adsorption process is more exothermic in CNTY as evident from the largely negative enthalpy values from Table S2. The entropy change is also more negative for CNTY reflecting more orderliness at the biosorbent-solution interphase.

### Effect of sample volume, regeneration and interference studies

Laboratory scale column studies were done to test the applicability of developed biosorbents for their sustainability to higher sample volume. A 1.5 g biosorbent was packed to 2 cm bed height in a glass column of 30 cm length with a diameter of 2 cm and was allowed to settle for a minimum of 2 hours to avoid air voids before the start of the experiment. A Cr(VI) concentration of 5 mg L^−1^ was prepared and the column was loaded with 50 mL of 5 mg L^−1^ at 5 mL min^−1^ flow rate and the eluate concentration was checked periodically for every 10 mL using ion chromatography. 50 mL of 5 mg L^−1^ Cr(VI) was completely adsorbed effectively. Subsequently, 300 mL of the Cr(VI) solution was loaded continuously and the heavy metal was adsorbed completely beyond which there was saturation owing to the non-availability of active adsorption sites^52^. A sample volume of 350 mL and 250 mL was adsorbed effectively onto CNTY and CNTR respectively as shown in Fig. S6a.

A significant property of the adsorbent is the ability to reuse thereby reducing the operational cost in treating pollutants. Varying sodium hydroxide concentrations (0.1–2.0 mol L^−1^) were tried (Fig. S6b,c) of which 1.0 mol L^−1^ sodium hydroxide was effective in desorbing Cr(VI) as sodium chromate^25^. It was observed that in CNTY, second, third, fourth, fifth cycles completed 100% adsorption- desorption cycles beyond which there was a decrease in the adsorption percentage. 70% adsorption was observed in 6^th^ cycle, 45% in 7^th^ cycle as shown in Fig. S6d,e. In CNTR, there were four complete adsorption-desorption cycles and the decrease in adsorption was observed from 5^th^ cycle.

A 100 mg L^−1^ concentration each of various cations such as Mn^+2^, Cu^+2^ Fe^+2^, Co^+2^, Ni^+2^, Pb^+2^ and anions such as nitrate, chloride, sulfate were added to 5 mg L^−1^ Cr(VI) solution and the adsorption studies were carried out to observe the influence of these ions in the uptake of chromium. It was observed that the adsorption percentage decreased by 2.0 ± 0.5% in CNTY as well as CNTR attributed to the interference of anions which compete with hydrochromate ions to occupy the adsorption sites, whereas cations such as Fe(II) and Mn (II) have the ability to reduce Cr(VI) to Cr(III)^53^.

## Discussion

The oxidised MWCNTs have larger surface area compared to the microbe immobilized carbon nanotubes. Also the pore parameters such as pore volume and pore diameter contribute to the surface area. In the current method the oxidised MWCNTs have a higher pore volume compared to microbe immobilized MWCNT. However for adsorption studies apart from the surface area, availability of more functional groups also aid in enhancing the metal uptake. The surface of the microbial cell wall have carboxyl, hydroxyl and amine functionalities which tend to participate in the metal uptake along with functionalised MWCNTs thereby increasing the Cr(VI) adsorption capacity.

Clearly yeast showed better adsorption capacity and the difference can be attributed to the morphological properties such as cell wall composition. The *Rhizobium* cell wall composition is similar to gram negative bacterial cell wall. Major component of cell wall is peptidoglycan which is made up of alanine, amino sugars, glutamic acid and diaminopimelic acid. It also contains lipo-polysaccharide which is composed of uronic acid, glucosamines, glucose, 2-keto, 3-deoxy octanoic acid, mannose and galactose which have functional groups such as amines, carboxyl, hydroxyl and phosphates^54^. The yeast cell wall composition is similar to gram positive bacteria which is majorly made up of polysaccharides such as β-glucans (60%), mannoproteins (40%), and chitin (2%)^55^ which mainly consists of amines, carboxylic and hydroxyl groups in larger amounts than in *Rhizobium* cell wall. Although, both the microbial cell walls are made up of polysaccharides, lipids and proteins, yeast cell wall has (1–10%) more lipid content^56^ owing to the presence of more functional groups. Hence, in the current work the uptake of chromium (VI) in yeast is higher as compared to *Rhizobium*.

When microbes are added as amine sources to the EDC-HOBT coupling reactions with oxidised MWCNTs, a covalent interaction in the form of amide bond is formed between them which participates in Cr(VI) adsorption in acidic medium and also the available OH, COOH groups in microbes augments the electrostatic interactions with the hydrochromate ion. The comparison of adsorption capacities of various adsorbents with the current developed biosorbents is given in Table S3. Clearly, the microbe immobilized MWCNTs shows a higher adsorption capacity as compared to the pristine and oxidised MWCNTs.

In conclusion, this work has highlighted the confluence of biotechnology and nano materials as an emerging area towards heavy metal remediation. The proposed methodology has illustrated the ability of two diverse microorganisms in oxidised multiwalled carbon nanotubes as effective adsorbents to sequester chromium in the +6 oxidation state. The biosorbents CNTR and CNTY followed Langmuir isotherm with 24.86 mg g^−1^ and 31.6 mg g^−1^ adsorption capacities respectively. The biosorption process was exothermic, spontaneous and pseudo second order model was effective in understanding the adsorption kinetics. The mechanism involves electrostatic interaction between the heavy metal ion and biosorbent surface. Characterisation techniques confirmed the interaction of microbes and oxidised carbon nanotubes with Cr(VI). A good sample volume of synthetic waste water sample was treated in lab scale column studies which could tolerate up to 4–5 cycles of adsorption and desorption by regenerating the biosorbents using 1.0 mol L^−1^ sodium hydroxide. It was observed that CNTY has better adsorption capacity than CNTR owing to the larger accessibility of functional groups present in the microbial cell walls. On an optimistic note, biotechnology and nanoscience complement each other by opening diverse possibilities in detoxifying the pollutants from industrial waste water.

## Materials and Methods

### Chemicals and materials

All the chemicals used were analytical and guaranteed reagents. MWCNTs type 5 were procured from Sisco Research Laboratories, India with an outer diameter of 30–50 nm and length 10–30 µm. A 1000 mg L^−1^ Cr(VI) stock solution was prepared from potassium dichromate (K_2_Cr_2_O_7_, Merck) and further dilutions were prepared accordingly in high purity Milli Q water. The solvents and salts used for Cr(VI) analysis using ion chromatography were supplied by Merck. The chemicals used to prepare YEPD medium (Yeast extract, peptone, dextrose) to culture yeast and YMA medium (Yeast extract, Mannitol, agar/broth) for the growth of rhizobium were procured from Himedia. The chemicals used in coupling reaction were (3-Dimethylaminopropyl)-N′-ethylcarbodiimide hydrochloride (EDC), Hydroxybenzotriazole (HOBT)(Sisco Research Laboratories, India), triethylamine (Merck), dimethylformamide (SD Fine Chemicals Ltd, India).

### Isolation and identification of microbial species

#### Rhizobium species

*Rhizobium* species was isolated from soil that was collected from nearby legume crop fields and followed a simple isolation procedure using standard pour plate technique on a YMA medium containing yeast extract, mannitol, NaCl, MgSO_4_, K_2_HPO_4_, Congo red and agar. The inoculated plates were incubated at 37 °C for 48 hours. Based on the morphological characteristics of the colonies, four isolates of the bacteria were selected (BI 1, BI 3, BI 4, BI 6). Further the isolates were subjected to morphological tests such as gram staining and motility tests, biochemical tests popularly known as IMVIC tests and molecular characterisations for the bacterial confirmation^57^.

#### Saccharomyces cerevisiae

The yeast granules purchased from local market were revived on YEPD medium and identified as *S*.*cerevisiae* through morphological and biochemical tests as reported by our group previously^25^.

### Genomic DNA isolation and 16S rDNA PCR amplification

The bacterial genomic DNA was isolated according to a standard DNA isolation protocol^58,59^. The strains isolated were grown in 5 mL YMA medium at 37 °C overnight and mid log phase of the culture obtained was harvested as a pellet by centrifugation. The pellet obtained was re-suspended in 500 µL of TEG buffer (Tris-50mM EDTA-50mM Glucose −20%) for the cell lysis. Further it was treated with lysozyme and RNAase at 37 °C for 40 min followed by action of 10% SDS solution at 37 °C for 60 min. Isolation was accomplished by adding equivalent quantities of Phenol, Chloroform and Isoamyl alcohol followed by precipitating with 90% isopropanol (ice cold). The white DNA precipitate which was obtained was washed with 70% ethyl alcohol and suspended in 30 µL TE (Tris-EDTA) buffer for further analysis.

The amplification of 16S rDNA was performed using Polymerase chain reaction (PCR). The PCR reaction was setup using 25 µL reaction mixture containing 200 µM dNTP, 1 µL of *Taq polymerase*, 10 × PCR buffer (2.5 µL) with MgCl_2_ (1.5 mM), 100 ng/µl genomic DNA, with 200 ng of forward [27 F 5′-AGAGTTTGATCMTGGCTCG-3′] and reverse primers [1492 R 5′-GGTTACCTTGTTACACTT-3′]^60^. The PCR program was operated starting with initial denaturation at 94 °C for 4 min, followed by denaturation for 1 min at 94 °C for 32 cycles, annealing at 58.5 °C for 50 min, extended for 2 min at 72 °C followed by a final extension at 72 °C for 10 min^61^ and the obtained product was resolved using electrophoresis. The purified amplicons were commercially sequenced. The sequences obtained were used to carry out a NCBI blast search analysis to confirm the identity of bacteria as *Rhizobium BVR*.

### Preparation of the *Rhizobium* – MWCNTs and *Yeast* – MWCNTs biosorbent

The isolated *Rhizobium* species was grown in yeast- mannitol broth medium and spun down to make a pellet. The yeast grown on YEPD medium was used^25^. Pristine MWCNTs were functionalised by covalent modification as reported previously^31,62^. A 200 mL of 0.5 mol L^−1^ H_2_SO_4_, was mixed with 0.25 g of KMnO_4_ as an initial step for oxidation. In another beaker, 0.1 g of MWCNTs, 200 mL of 0.5 mol L^−1^ H_2_SO_4_ was added and subjected to ultra-sonication for 30 min to ensure proper dispersion. After sonication the solution containing MWCNTs was heated up to 150 °C prior to the addition of KMnO_4_ solution dropwise. The mixed solution was refluxed for 5 hours at 150 °C and cooled down to room temperature proceeded by the addition of 10 mL of concentrated HCl to dissolve MnO_2_. The oxidised MWCNTs were washed till the pH reached between 6.0–7.0 and then dried at 100 °C.

The dried MWCNTs were further used in the preparation of biosorbent along with microbes (free amine sources) by involving in EDC-HOBT coupling. To 0.1 g of oxidised MWCNTs, 0.1 g each of EDC, HOBT were added. The solvent used was DMF (dimethyl formamide) and 3 mL of triethylamine was added to the above mixture and stirred for 20 minutes. 3.0 g of yeast was added to the solution and the mixture was stirred overnight for the coupling reaction to take place^63^. Similar procedure was repeated for the coupling reaction with *Rhizobium BVR*. The coupling mixture was filtered, washed with water and dried at 80 °C for 4 hours before proceeding for metal adsorption studies.

### Synthesis of probes for Cr(VI) and Cr(III)

Cr(VI) and Cr(III) have specific binding probes to differentiate them. Rhodamine based sensors are selected due to its spiro lactam structure and spiro ring opening of sensing a molecule. Rhodamine B hydrazide (RBH)^45^ and Rhodamine based chemo sensor (RF)^46^ were synthesized as described in literature which are specific for Cr(VI) and Cr(III) respectively and the samples were prepared for capturing the laser confocal images.

### Characterisation techniques

The developed biosorbent was characterized using various analytical techniques before and after Cr(VI) adsorption. FTIR analysis was performed for the Pristine CNT, microbe-carbon adsorbent (before as well as after adsorption) using JASCO– 4200 model spectrometer (400–4000 cm^−1^) where 1.0 mg of the samples were mixed with 100 mg of KBr and the individual spectra were recorded. Thermogravimetric analysis (TGA) of the biosorbent was recorded using a Shimadzu DTG-60 thermal analyser. A known weight of the biosorbent was taken and subjected to a temperature range of 30–800 °C under air atmosphere to analyze the thermal stability. For the elemental analysis energy dispersive X-ray spectra (EDAX) was recorded using Bruker – X Flash 6/30 and to study the surface morphology of the biosorbents, field emission scanning electron microscopic (FESEM) images were captured using Carl Zeiss Supra 55 and High resolution transmission electron microscopic (HRTEM) images taken by Tecnai 20 (FEI) 200 kV gives an insight into the microstructure and the defects at atomic resolution. The elemental speciation was ascertained by X-ray photo electron spectroscopy using PHI 5000 Versa Prob II, FEI Inc, and the source used is an aluminium monochromator at 25.4 W and 187.85 eV. The BET surface area, BJH pore volume and average pore diameter of the biosorbent were measured using a BELSORP II mini (Microtrac BEL Corp) at an outgassing temperature of 150 °C for 2 hour duration. LeicaDMi8 laser scanning microscopy (S/N 418513) was used to capture confocal images. The quantitative Cr(VI) adsorption was performed using 883 Basic IC plus Ion chromatography^64^ with a 887 professional UV/Vis detector. The 16S rDNA sequencing was carried out using the Biosystems ABI 3730 xls Genetic analyser at Bioserve Pvt. Ltd., Hyderabad, India. Matrix assisted laser desorption ionisation time of flight (MALDI-TOF) was performed using VITEK MS (Biomerieux) spectrometer, with the knowledge Base v2 database (closed) at Zeal Biologicals, Hyderabad, India for the confirmation of the isolated bacteria.

### Batch adsorption studies

The batch adsorption studies were performed by optimizing various parameters such as pH, adsorbent dosage, kinetics, thermodynamics. A 0.1 g of the biosorbent was used to treat 20 mL of 5 mg L^−1^ Cr(VI) solution at pH 2.0. The equilibration was performed for 180 min using an orbital incubator shaker (Biotechnics, India) operating at 120 rpm maintained at 30 °C. After separation of the biosorbent through filtration, the concentration of Cr(VI) was estimated using ion chromatography coupled with a UV detector and the amount of Cr(VI) adsorbed onto the developed biosorbents was calculated as

4 $${{\rm{q}}}_{{\rm{e}}}=\frac{({{\rm{C}}}_{{\rm{o}}}-{{\rm{C}}}_{{\rm{e}}}){\rm{V}}}{{\rm{W}}}$$

q_e_ = amount of Cr(VI) adsorbed (mg g^−1^) onto biosorbent. C_o_ = initial Cr(VI) concentration. Ce = Cr(VI) concentration remaining in solution phase. V = Volume of Cr(VI) solution used for biosorption (L). W = weight of the biosorbent used for Cr(VI) treatment (g).

## Electronic supplementary material

Supplementary Information
