# Supplementary material for: Potential Application of Saccharomyces cerevisiae and Rhizobium Immobilized in Multi Walled Carbon Nanotubes to Adsorb Hexavalent Chromium
Source: Sci Rep. 2018 Jun 29;8:9862. doi: 10.1038/s41598-018-28067-9 (PMC6026182; doi:10.1038/s41598-018-28067-9)
Supplement: Supplementary file 1 — Supplementary Information [file 41598_2018_28067_MOESM1_ESM.docx]

*Electronic Supplementary Information for*

**Potential Application of *Saccharomyces cerevisiae* and *Rhizobium* Immobilized in Multi Walled Carbon Nanotubes to Adsorb Hexavalent Chromium**

T. Sathvika^1^, Amitesh Soni^1^, Kriti Sharma^1^, Malipeddi Praneeth^1^, Manasi Mudaliyar^2^, Vidya Rajesh^2^ and N. Rajesh^1*^


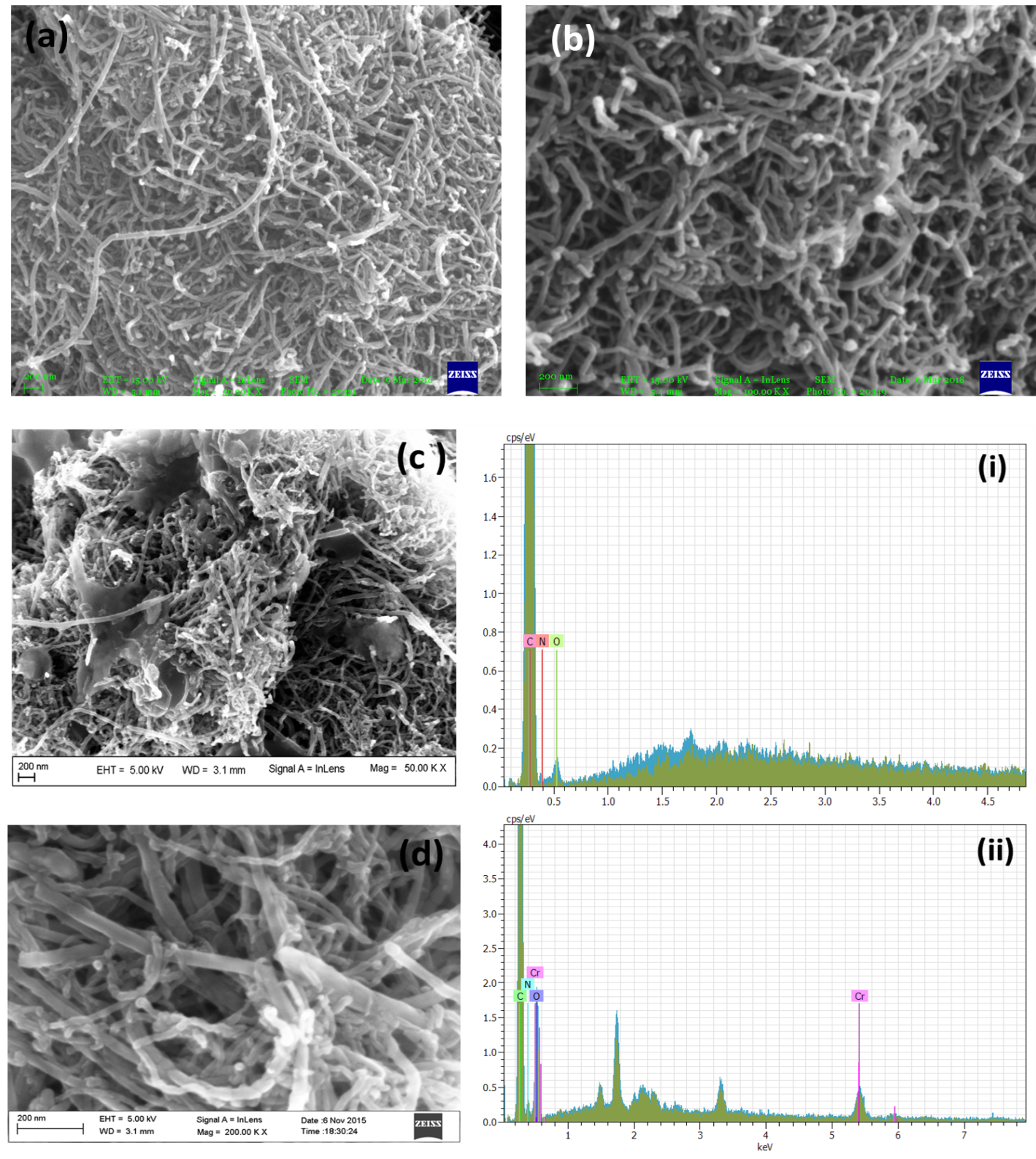


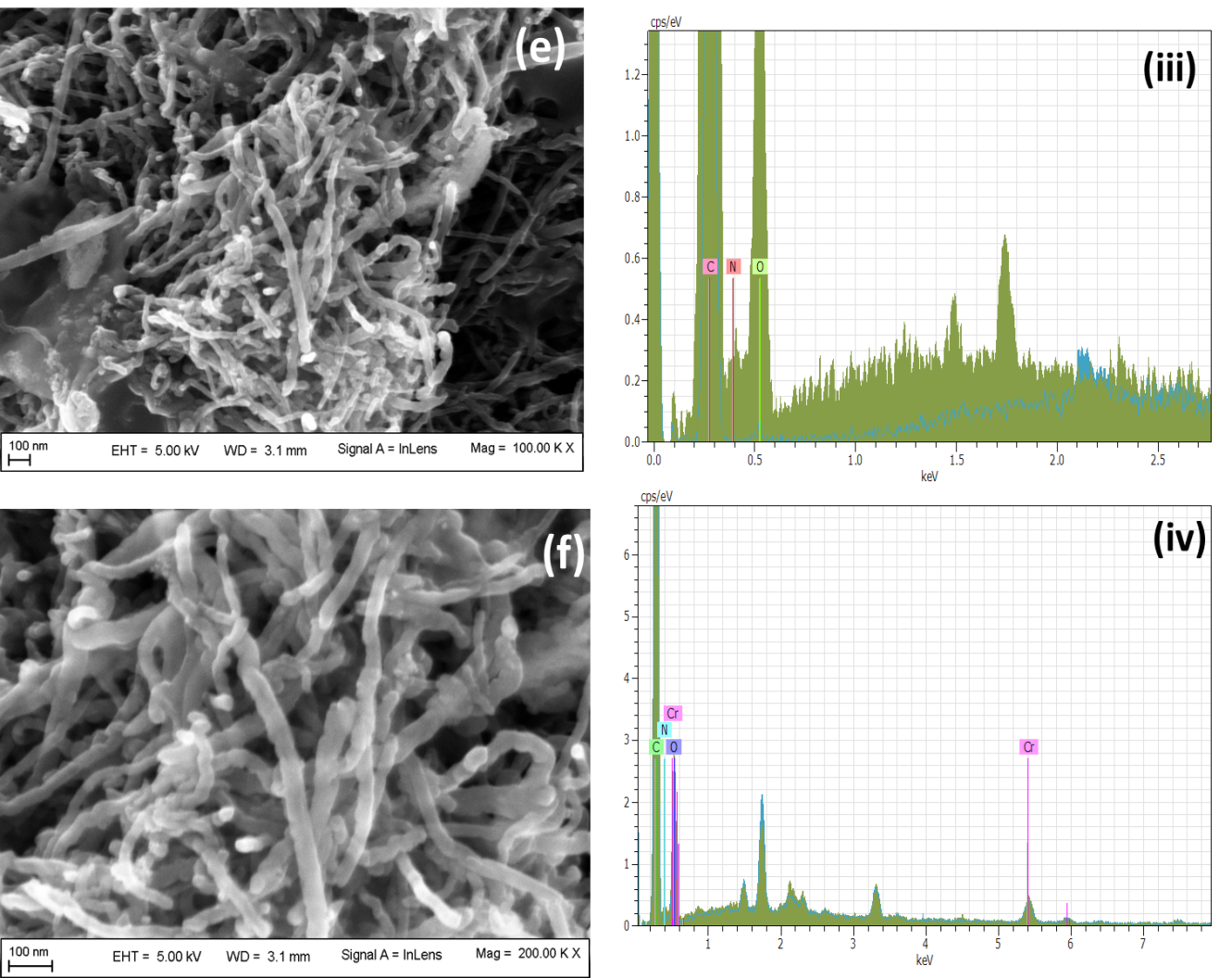


**Figure S1.** FESEM images of (a) pristine MWCNTs

(b) oxidized MWCNTs

(c,d) CNTR before and after Cr(VI) adsorption respectively

(e,f) CNTY before and after Cr(VI) adsorption.

EDAX spectra of (i, ii) CNTR before and after Cr(VI) adsorption

(iii,iv) CNTY before and after Cr(VI) adsorption.


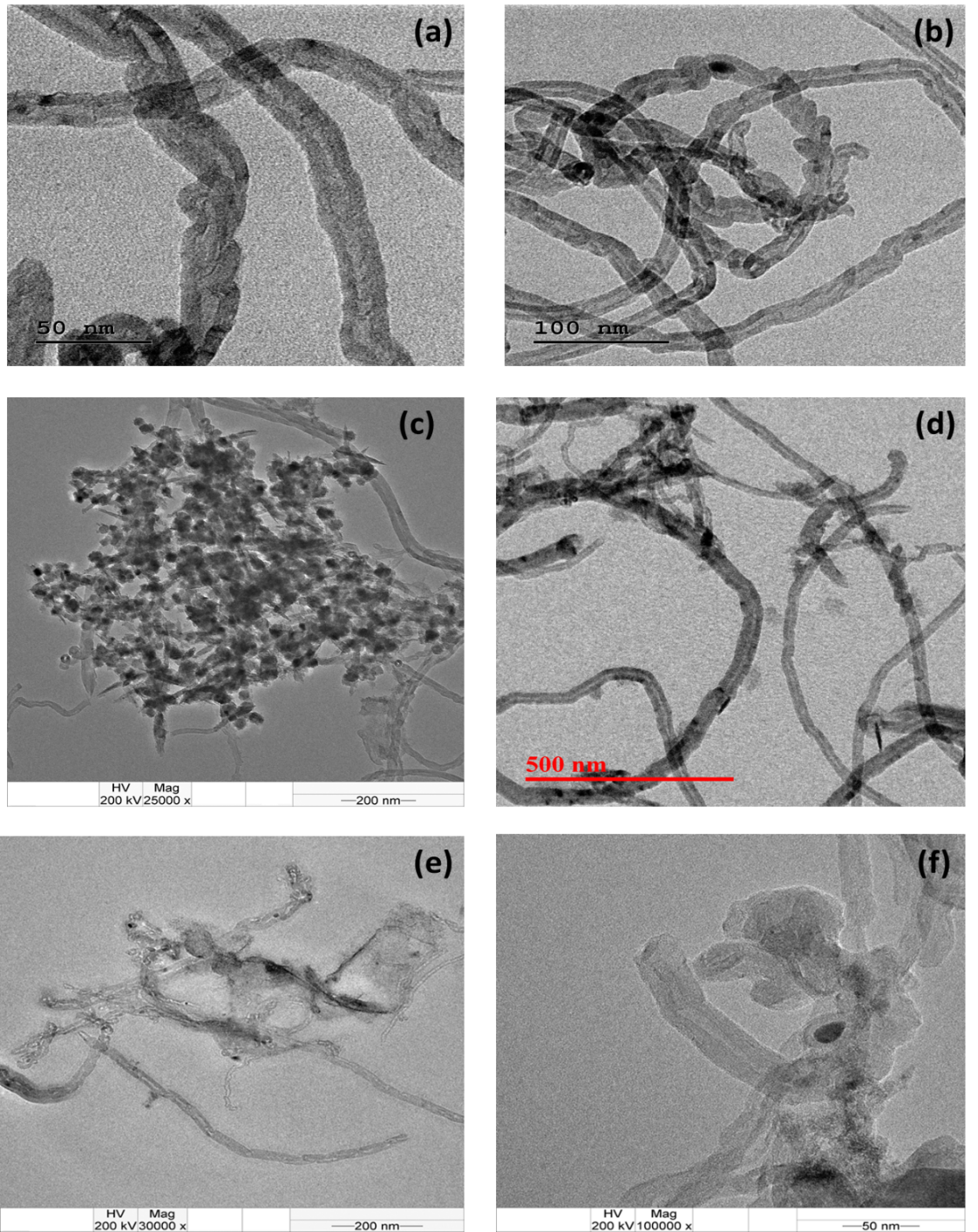


**Figure S2.** HRTEM images of (a) pristine MWCNTs

(b) oxidized MWCNTs

(c,d) CNTR before and after Cr(VI) adsorption

(e,f) CNTY before and after Cr(VI) adsorption.


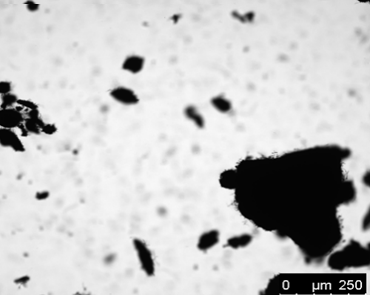

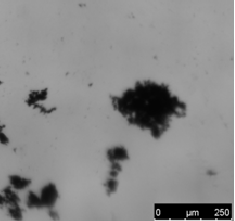

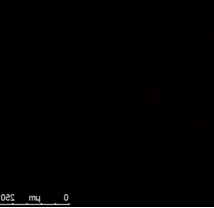

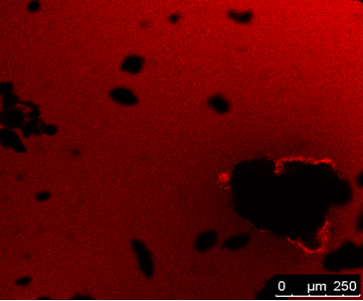


**a**

**b**

**c**

**d**


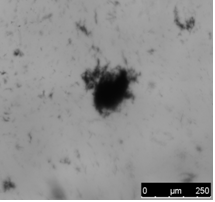

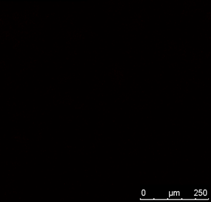

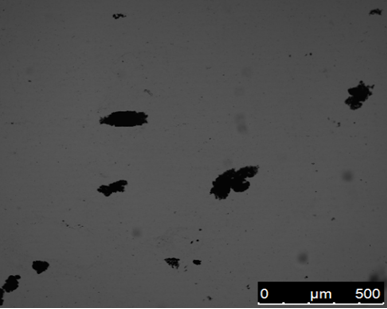

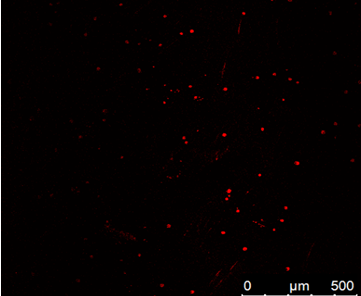


**e**

**f**

**g**

**h**


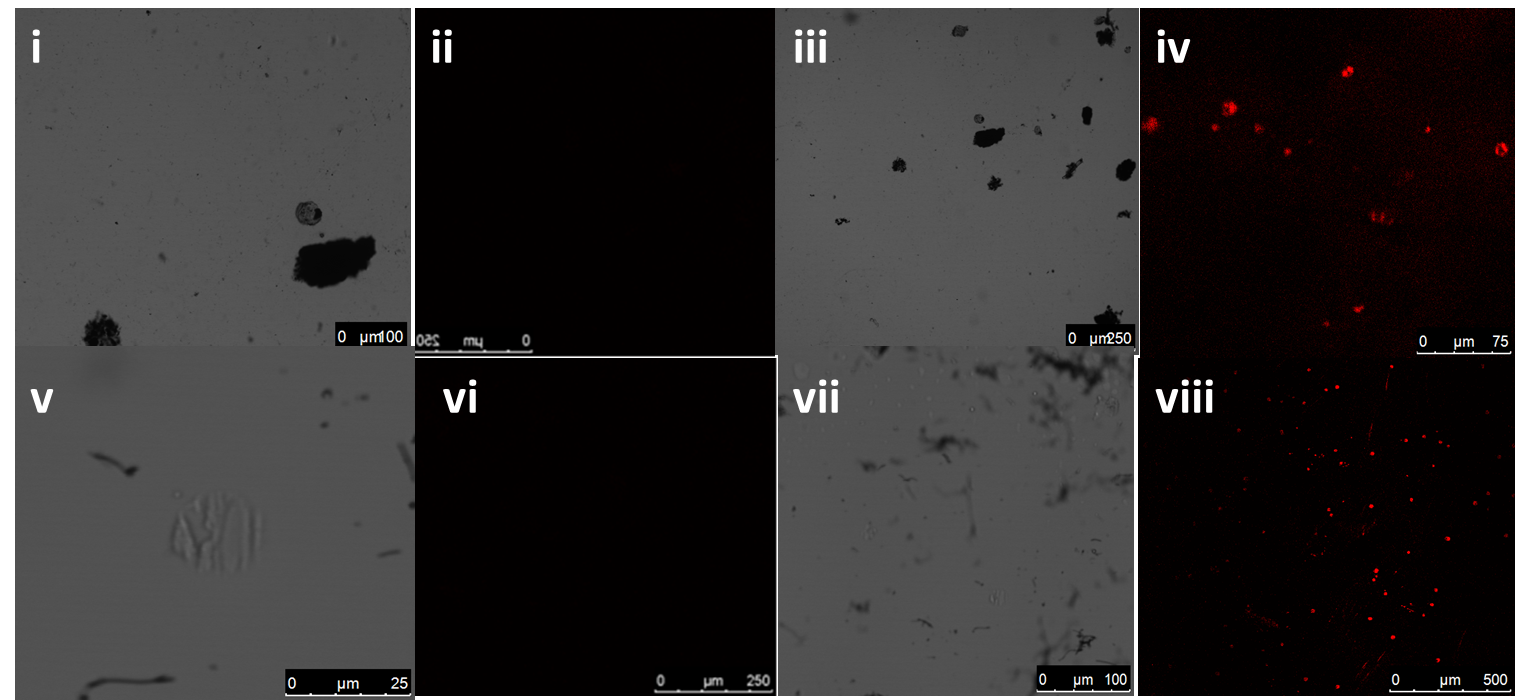


**Figure S3.** Confocal and bright field images of the bio sorbent after Cr(VI) adsorption: (a,b) CNTR with RBH (c,d) CNTR after Cr(VI) adsorption with RBH (e,f) CNTR with RF (g,h) Cr(VI) reduction to Cr(III) on CNTR with RF.

(i,ii) CNTY with RBH (iii,iv) CNTY after Cr(VI) adsorption with RBH (v,vi) CNTY with RF (vii,viii) Cr(VI) reduction to Cr(III) on CNTY with RF.

**Figure S4.** (a) pH effect on bio sorption of CNTY and CNTR (b) Effect of adsorbent dosage on biosorption of CNTY, CNTR

**
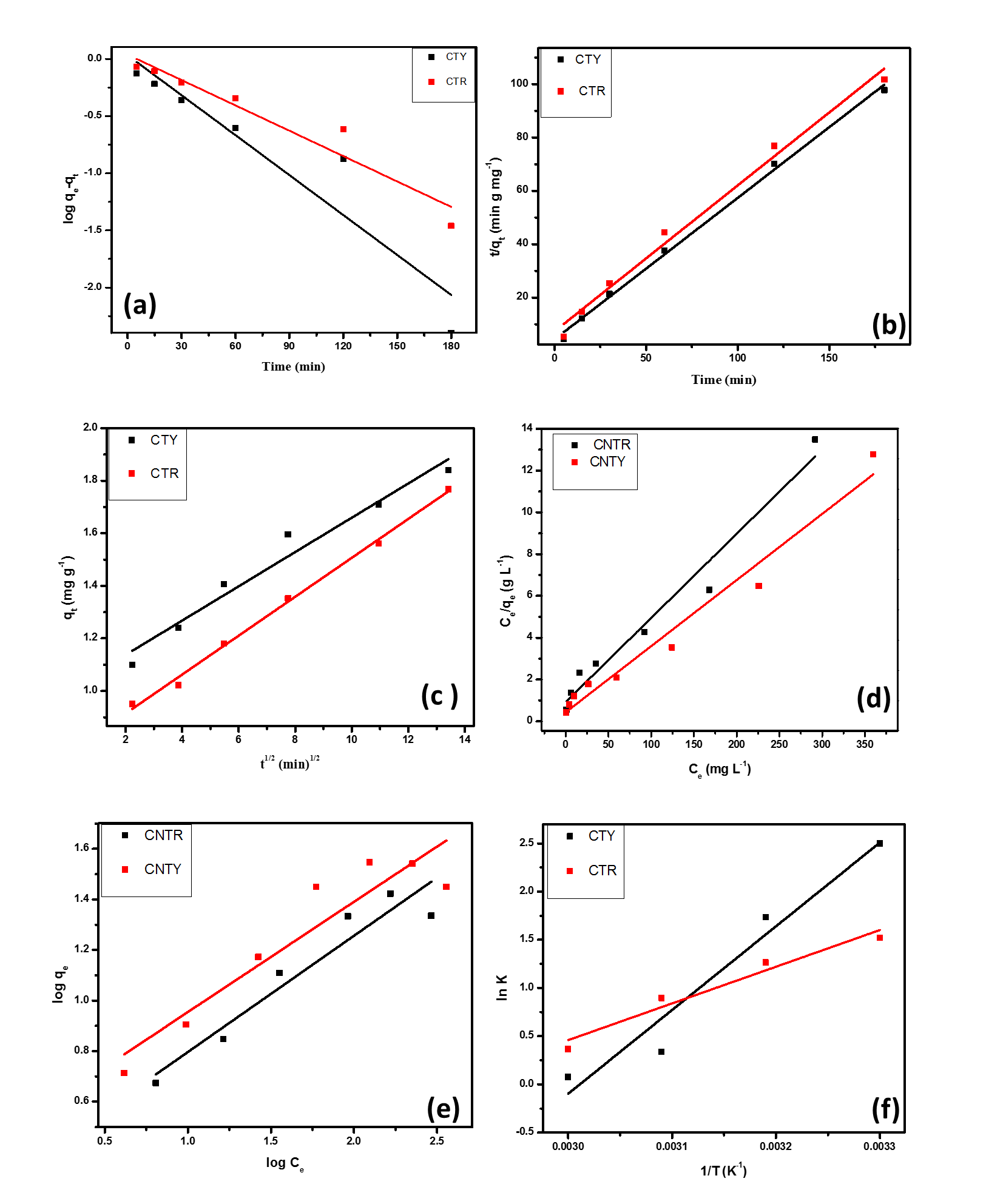
**

**Figure S5.** (a) Pseudo first order kinetics (b) pseudo second order kinetics (c) Intra particular diffusion (d) Langmuir (e) Freundlich (f) lnK against 1/T


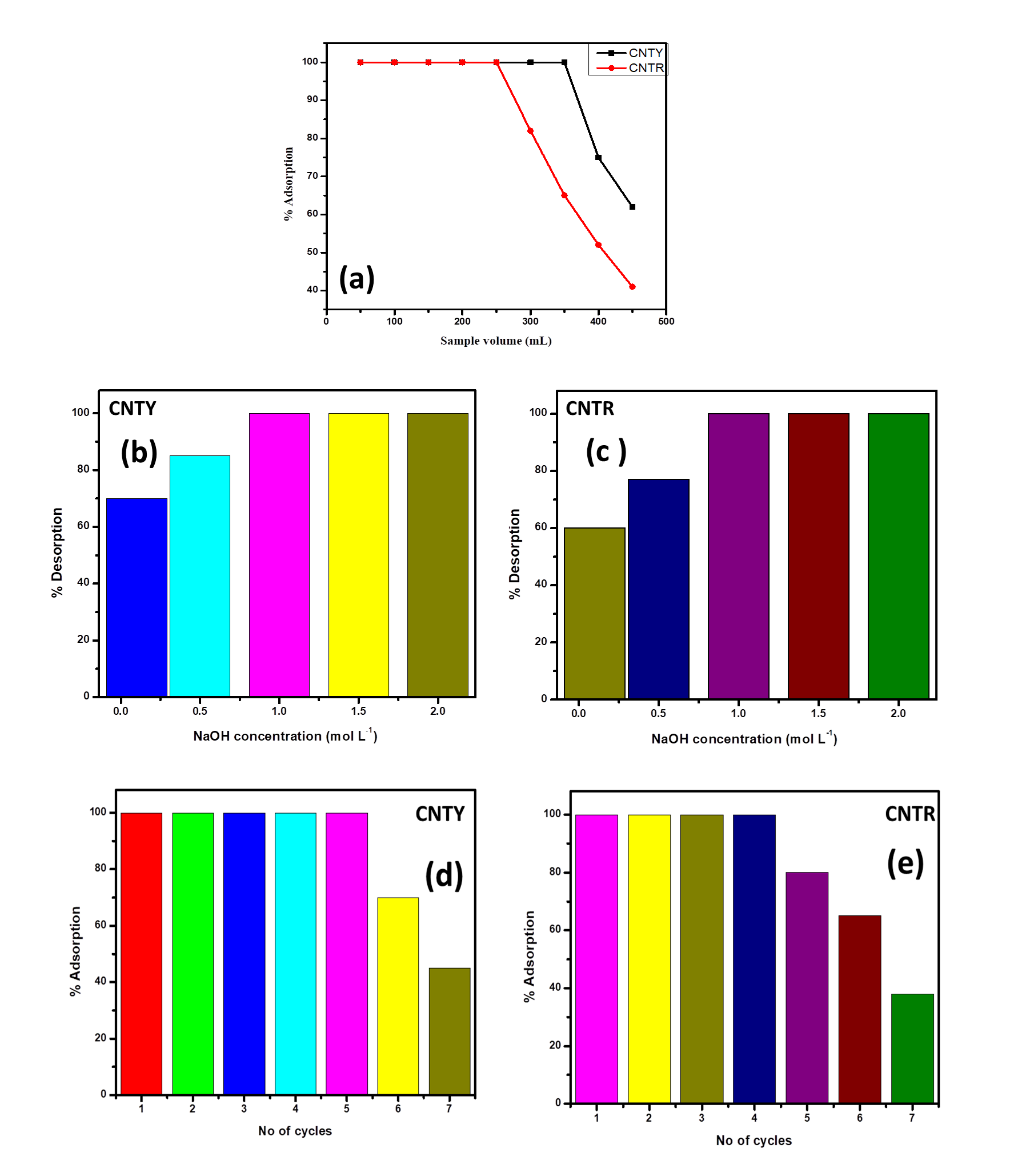


**Figure S6**. (a) Effect of sample volume of CNTR and CNTY (b,c) Effect of varied NaOH concentrations on CNTR, CNTY (d,e) Regeneration efficiency of CNTR and CNTY

| **Kinetic parameters** |  | **CNTY** |  | **CNTR** |
| --- | --- | --- | --- | --- |
|  |  |  |  |  |
| C_o_ (mg L^-1^) |  | 10 |  | 10 |
| q_e_ (mg g^-1^) |  | 1.884 |  | 1.826 |
| k_2_ (g mg^-1^ min^-1^) |  | 0.064 |  | 0.041 |
| R^2^ |  | 0.996 |  | 0.986 |
| k_1_ (min^-1^) |  | 0.026 |  | 0.017 |
| R_1_^2^ |  | 0.868 |  | 0.915 |
| k_int_ (mg g^-1^ min^-0.5^) |  | 0.065 |  | 0.074 |

**Table S1**The kinetic parameters for the adsorption of Cr(VI) onto CNTY and CNTR

| **CNTY** | **CNTR** | |
| --- | --- | --- |
|  |  | |
| **T ΔG^o^ ΔS^o^ ΔH^o^ E_a_**  **(Kelvin) (kJ mol^-1^) (J mol^-1^ K^-1^) (kJ mol^-1^) (kJ mol^-1^)** |  | **T ΔG^o^ ΔS^o^ ΔH^o^ E_a_**  **(Kelvin) (kJ mol^-1^) (J mol^-1^ K^-1^) (kJ mol^-1^) (kJ mol^-1^)** |
|  |  |  |
| 303 -6.29  313 -4.513 -217.3 -72.2 -69.55  323 -0.907  333 -0.2187 |  | 303 -3.705  313 -3.186 -91.1 -31.6 -29.0  323 -2.329  333 -0.977 |

**Table S2** Thermodynamic parameters associated with adsorption

| Adsorbents | pH | Adsorption  capacity (mg g^-1^) |
| --- | --- | --- |
| Cellulose-sodium montmorillonite^53^ | 3.8-5.5 | 22.2 |
| MWCNTs –calcium alginate complex immobilized in *Shewanellaoneidensis*^35^ | 6.0-7.0 | 6.07 |
| Fe_2_O_3_ nanoparticle-MWCNTs composite^32^ | 2.0 | 42.02 |
| Activated carbon supported MWCNTs^31^ | 2.0 | 113.29 |
| (Present studies)  *Rhizobium BVR*  *Saccharomyces cerevisiae*  Pristine MWCNTs  Oxidized MWCNTs  CNTR  CNTY | 2.0 | 9.50  11.2  11.93  16.22  24.82  31.6 |

**Table S3** Comparison of adsorption capacities of various biosorbents
